# Symbiont strain is the main determinant of variation in *Wolbachia*‐mediated protection against viruses across *Drosophila* species

**DOI:** 10.1111/mec.14164

**Published:** 2017-05-30

**Authors:** Julien Martinez, Ignacio Tolosana, Suzan Ok, Sophie Smith, Kiana Snoeck, Jonathan P. Day, Francis M. Jiggins

**Affiliations:** ^1^ Department of Genetics University of Cambridge Cambridge UK

**Keywords:** *Drosophila*, symbiont‐mediated protection, viruses, *Wolbachia*

## Abstract

*Wolbachia* is a common heritable bacterial symbiont in insects. Its evolutionary success lies in the diverse phenotypic effects it has on its hosts coupled to its propensity to move between host species over evolutionary timescales. In a survey of natural host–symbiont associations in a range of *Drosophila* species, we found that 10 of 16 *Wolbachia* strains protected their hosts against viral infection. By moving *Wolbachia* strains between host species, we found that the symbiont genome had a much greater influence on the level of antiviral protection than the host genome. The reason for this was that the level of protection depended on the density of the symbiont in host tissues, and *Wolbachia* rather than the host‐controlled density. The finding that virus resistance and symbiont density are largely under the control of symbiont genes in this system has important implications both for the evolution of these traits and for public health programmes using *Wolbachia* to prevent mosquitoes from transmitting disease.

## 1 INTRODUCTION

*Wolbachia* is a maternally transmitted bacterial symbiont that produces a remarkably diverse array of phenotypes in arthropods. In many cases, it manipulates its host's reproduction to increase its transmission to future generations, for example, by distorting sex ratios or inducing cytoplasmic incompatibility (CI) (Werren, Baldo, & Clark, 2008). More recently, it was discovered that many *Wolbachia* strains can protect their hosts against viral pathogens (Hedges, Brownlie, O'Neill, & Johnson, 2008; Teixeira, Ferreira, & Ashburner, 2008). Other *Wolbachia* infections have been associated with an array of other phenotypes, ranging from being mutualists that synthesize essential nutrients (Hosokawa, Koga, Kikuchi, Meng, & Fukatsu, 2010) to causing reductions in survival and fecundity (Martinez et al., 2015).

This phenotypic variation across host–*Wolbachia* associations could be caused by genetic differences in the hosts, the symbionts or both partners. Understanding the determinants of this variation is important because, over evolutionary timescales, *Wolbachia* jumps between host species (Vavre, Fleury, Lepetit, Fouillet, & Boulétreau, 1999; Werren, Zhang, & Guo, 1995; Zhang, Han, & Hong, 2013). Whether a phenotype is controlled by the host or the symbiont genome will determine if *Wolbachia*‐induced phenotypes are transferred along with the infection to the new host and therefore affect the success of the host shift. From an applied perspective, artificially moving the bacterium between host species allows *Wolbachia* to be used as a biocontrol agent. Strains of *Wolbachia* have been transferred from *Drosophila* to the mosquito *Aedes aegypti* with the aim of preventing the transmission of dengue virus (Frentiu et al., 2014; Joubert et al., 2016; Moreira et al., 2009; Walker et al., 2011; Yeap et al., 2011). Understanding what governs changes in phenotype following a host shift can thus help predict the success of such symbiont‐based applications, and will determine whether model species like *Drosophila melanogaster* can be used to identify the best symbiont strains to transfer to mosquitoes.

The role of the host genome in determining the phenotype of *Wolbachia* infections has been investigated by experimentally moving *Wolbachia* between host species. Many of these studies have investigated reproductive manipulations such as cytoplasmic incompatibility and sex ratio distortion (Fujii, Kageyama, Hoshizaki, Ishikawa, & Sasaki, 2001; Jaenike, 2007; Poinsot, Bourtzis, Markakis, & Savakis, 1998; Sakamoto et al., 2005; Veneti et al., 2012). Here, host shifts have been shown to be associated with changes in the intensity of the phenotype (Poinsot et al., 1998), a complete loss of the phenotype (Veneti et al., 2012) or even a switch in the type of reproductive alteration (Jaenike, 2007).

The roles of host and symbiont genomes in determining whether *Wolbachia* blocks viral replication are especially important as considerable effort is being put into transferring symbiont strains to mosquitoes to prevent the transmission of viral pathogens (Hoffmann, Ross, & Rašić, 2015). The antiviral phenotype of *Wolbachia* was first observed in *D. melanogaster* (Hedges et al., 2008; Teixeira et al., 2008), and later in other *Drosophila* species (Cattel, Martinez, Jiggins, Mouton, & Gibert, 2016; Osborne, Leong, O'Neill, & Johnson, 2009; Unckless & Jaenike, 2011) and mosquitoes (Bian, Zhou, Lu, & Xi, 2013; Blagrove, Arias‐Goeta, Failloux, & Sinkins, 2012; Glaser & Meola, 2010; Moreira et al., 2009). The ability of *Wolbachia* to spread by manipulating host reproduction in combination with its antiviral properties makes it a promising tool for the control of mosquito‐borne viruses like dengue and Zika (Aliota, Peinado, Velez, & Osorio, 2016; Dutra et al., 2016; Moreira et al., 2009). Currently, large‐scale field trials are evaluating whether releasing *Ae. aegypti* mosquitoes infected with a *Wolbachia* strain from *D. melanogaster* prevents dengue transmission (Frentiu et al., 2014; Hoffmann et al., 2011). There is extensive genetic variation among symbiont strains in the level of antiviral protection (Bian et al., 2013; Blagrove et al., 2012; Chrostek, Marialva, Yamada, O'Neill, & Teixeira, 2014; Chrostek et al., 2013; Martinez et al., 2014; Osborne et al., 2009). However, little is known about the role of the host genotype in affecting this trait. Two strains of *Wolbachia* have been transferred from *D. melanogaster* to *Ae. aegypti*, and in both host species, an over‐replicating laboratory mutant called *w*MelPop had the strongest antiviral effects (Chrostek et al., 2013; van den Hurk et al., 2012; Hussain et al., 2013). In a different system, moving a *Spiroplasma* symbiont between *Drosophila* species determined whether it protected its host against parasitic nematodes (Haselkorn, Cockburn, Hamilton, Perlman, & Jaenike, 2013).

Here, we compared *Wolbachia* strains in their native host and a new host to test whether the host and/or symbiont genome determines the level of antiviral protection. We first assessed the frequency of antiviral protection in 16 natural host–symbiont associations. We then compared the level of protection induced by eight of these *Wolbachia* strains in both their original host and a line of *D. simulans* to which they have been artificially transferred. We find that the level of antiviral protection is largely determined by the *Wolbachia* strain rather than the host species.

## 2 METHODS

### 2.1 *Drosophila* stocks and *Wolbachia* strains

All *Drosophila* species were maintained on a cornmeal diet (see recipe in Longdon et al., 2015) at 25°C, under a 12‐hr light/dark cycle and 70% relative humidity. Ten *Drosophila* species infected with their native *Wolbachia* strains were used in this study (Tables 1 and S1). Of these, more than one line of *D. melanogaster* and *D. simulans* was used, each infected with a different *Wolbachia* strain. For each *Wolbachia*‐infected fly line, we had a matching *Wolbachia*‐free control. *Wolbachia*‐infected *D. melanogaster* and the uninfected control were created using balancer chromosomes to homogenize their nuclear background as described in Chrostek et al. (2013). For all the other fly lines, a *Wolbachia*‐free line was created from *Wolbachia*‐infected flies by raising them on Ready Mix Dried Food (Philip Harris) supplemented with 0.03% w/v tetracycline for two generations. In order to homogenize the gut microbiota between *Wolbachia*‐infected lines and their tetracycline‐treated counterparts, the tetracycline‐treated lines were then raised for one generation on standard cornmeal food on which ten males of the respective *Wolbachia*‐infected line had been kept for 1 day and removed (as in Martinez et al., 2016). Experiments were all performed more than twenty generations after tetracycline treatment. The *Wolbachia* infection status of all fly lines was checked by PCR and Sanger sequencing as described below.

**Table 1:** Natural *Drosophila*–*Wolbachia* associations used in this study

*Drosophila* group	*Drosophila* subgroup	*Drosophila* species	*Wolbachia* strain
*melanogaster*	*ananassae*	*D. ananassae*	*w*Ana^a^
	*melanogaster*	*D. melanogaster*	*w*MelCS^a^
		*D. melanogaster*	*w*MelPop
		*D. melanogaster*	*w*Mel^a^
		*D. sechellia*	*w*Sh^a^
		*D. simulans*	*w*Ha
		*D. simulans*	*w*Ma
		*D. simulans*	*w*No
		*D. simulans*	*w*Au^a^
		*D. simulans*	*w*Ri
		*D. teissieri*	*w*Tei^a^
	*montium*	*D. triauraria*	*w*Tri
	*suzukii*	*D. suzukii*	*w*Suz
*saltans*	*saltans*	*D. prosaltans*	*w*Pro^a^
	*sturtevanti*	*D. sturtevanti*	*w*Stv
*willistoni*	*willistoni*	*D. tropicalis*	*w*Tro^a^

a *Wolbachia* strains that were also used in the *D. simulans* line STCP (see Methods).

In order to compare the *Wolbachia* strains in their original host and in a new host, we also used the *D. simulans* line STCP into which some of the *Wolbachia* strains were previously transferred through backcrossing or microinjection (Martinez et al., 2014; Poinsot et al., 1998; Zabalou et al., 2008; Table S1). In order to minimize inbreeding depression, before each experiment STCP females were crossed to males of a different *Wolbachia*‐free isofemale line (14021–0251.175, Dsim\wild‐type, San Diego Drosophila Species Stock Center). All measurements were carried out on the emerging F1 adults from this cross, as in Martinez et al. (2015).

### 2.2 Virus production

To test antiviral protection, we used Flock House virus, which has a positive‐sense single‐stranded RNA genome. FHV belongs to the family Nodaviridae and was initially isolated from a beetle (Scotti, Dearing, & Mossop, 1983). We chose to use FHV instead of a native virus such as *Drosophila* C virus (Comendador et al., 1986; Plus, Croizier, Jousset, & David, 1975) as we have found that there is less genetic variation among hosts in susceptibility to FHV (Magwire et al., 2012). FHV was produced in Schneider Drosophila line 2 (DL2) cells. Cells were cultured at 26.5°C in Schneider's Drosophila medium with 10% foetal bovine serum, 100 U/ml penicillin and 100 mg/ml streptomycin (all Invitrogen, UK). Cells were then freeze‐thawed twice to lyse cells and centrifuged at 4,000 *g* for 10 min at 4°C to remove cellular debris. Virus was then aliquoted and frozen at −80°C. Virus infectivity was calculated using serial dilutions of virus in Schneider's medium added to wells of a plate of DL2 cells as described in Longdon, Cao, Martinez, and Jiggins (2013). After 7 days, the wells were visually examined under the microscope and classed as “infected” when cell death (presence of cell debris) and cytopathic effects were visible (lysing, shrinking or losing of compartmentation of cells). The Tissue Culture Infective Dose 50 (TCID50) was calculated by the Reed–Muench endpoint method (Reed & Muench, 1938).

### 2.3 *Wolbachia* screening

The *Wolbachia* infection status of fly lines was checked by PCR using the diagnostic primers wsp81F and wsp691R (Zhou, Rousset, & O'Neill, 1998). DNA from ten female flies per fly line was first extracted by crushing the flies in 150 μl of a 5% w/v suspension of Chelex 100 resin (Sigma‐Aldrich) and 1 μl of proteinase K (20 mg/ml, Fermentas). Extracts were incubated for 5 hr at 56°C. After 10 min at 95°C, samples were centrifuged and stored at −20°C. PCR conditions were as described in Ref. (Zhou et al., 1998). For the *Wolbachia*‐infected lines, the PCR products of the genes *wsp* and 16S (16Swol F: 5′‐TTGTAGCCTGCTATGGTATAACT‐3′; 16SWol R: 5′‐GAATAGGTATGATTTTCATGT‐3′, O'Neill, Giordano, Colbert, Karr, & Robertson, 1992) were Sanger‐sequenced to identify the *Wolbachia* infections at the strain level.

### 2.4 Survival assay

To infect flies with FHV, 3‐ to 6‐day‐old females were anaesthetized with CO_2_ and then stabbed into the left pleural suture of the thorax with a 0.15‐mm‐diameter anodized steel needle (Austerlitz Insect Pins) bent 0.25 mm from the end (half of the dorsal width of the thorax). The needle was either dipped into viral suspension or with a control solution produced from a virus‐free cell culture medium. The FHV stock was defrosted on the day of infection and then disposed of. Following infection, replicates of fifteen to twenty flies were placed in vials with standard cornmeal food. Survival was recorded every day. Flies were transferred into a fresh vial of food every 3 days.

Our first survival experiment was performed using all the *Wolbachia* strains in their original host species or background (i.e., not including the *Wolbachia* strains transferred into the *D. simulans* STCP line) and a virus dose of 3.6 × 10^10^ TCID_50_/ml. In this experiment, flies were placed at 22°C following virus infection in order to minimize the mortality that occurs in mock‐infected controls for some of the species (based on a pilot experiment). In a second experiment, eight of the *Wolbachia* strains were compared in parallel in their original host line and in the *D. simulans* line STCP (outcrossed as explained above). In this second experiment, flies were kept at 25°C following virus infection and the virus dose used was 3.6 × 10^8^ TCID_50_/ml. In both experiments, infections were carried out over 5–9 days. On each day, one biological replicate (vial of flies) per treatment (virus/mock infection, *Wolbachia*‐infected/uninfected, host line) was infected. The order of treatments was randomized between days. In total, five vials of flies were prepared for each treatment.

### 2.5 *Wolbachia* density

To measure the *Wolbachia* density within fly tissues, DNA was extracted using the Gentra Puregene kit (Qiagen) from a pool of ten 2‐ to 5‐day‐old females reared at 25°C. Flies from each *Wolbachia*‐infected fly line were collected every day from the same cohorts used in the second survival experiment. Five biological replicates (independent pools of females) were extracted for each *Wolbachia*‐infected line and the DNA was then diluted 1:10 with nuclease‐free water. For each *Drosophila* species, we sequenced the fly gene *RpL32* as in Longdon, Hadfield, Webster, Obbard, and Jiggins (2011) and designed species‐specific primers in two conserved regions for quantitative PCR (qPCR) (Table S2). The copy number of the *Wolbachia* gene *atpD* (atpDQALL_F: 5′‐CCTTATCTTAAAGGAGGAAA‐3′; atpDQALL_R: 5′‐AATCCTTTATGAGCTTTTGC‐3′) relative to the endogenous *Drosophila* control gene *RpL32* (species‐specific primers; Table S2) was quantified with the SensiFAST SYBR and Fluorescein kit (Bioline). For each gene, all samples were run on the same qPCR plate and a second technical replicate was performed on a different plate. The efficiency with which each set of primers amplified the product was checked using a dilution series. In all cases, the efficiency was >95% (with 100% efficiency equating to a doubling of the PCR product concentration every cycle). The *Wolbachia* density was estimated as 2^Δ*Ct*^, where *Ct* is the mean cycle threshold of the two technical replicates and Δ*Ct *=* Ct*_*RpL32*_ **−* Ct*_*atpD*_. The qPCR cycle was 95°C for 2 min, followed by 40 cycles of 95°C for 5 s and 60°C for 30 s.

### 2.6 Viral titre

In order to estimate FHV titre, flies were raised and females were infected with virus under the same conditions as the survival experiments. As the first experiment was performed at 22°C and the second one at 25°C, flies were collected 5 and 3 days post‐infection, respectively, in order to allow sufficient time for the viral replication before any significant mortality occurs. Flies were snap‐frozen in liquid nitrogen in pools of ten females (five to ten biological replicates from separate pools of flies per fly line). Flies were then homogenized in TRIzol. Total RNA was extracted using TRIzol (Invitrogen) and reverse‐transcribed with Promega GoScript reverse transcriptase (Promega) and random hexamer primers, and then diluted 1:10 with nuclease‐free water. The FHV RNA copy number (forward: 5′‐ACCTCGATGGCAGGGTTT‐3′; reverse: 5′‐CTTGAACCATGGCCTTTTG‐3′) relative to the endogenous control gene *RpL32* (species‐specific primers; Table S2) was quantified as for the *Wolbachia* density with two technical replicates per sample. As for the *Wolbachia* density, the species‐specific primers for *RpL32* were designed in two conserved regions, except that the forward primer was designed on an exon–exon junction in order to amplify only mRNA. This exon–exon junction was previously confirmed in several *Drosophila* species (Longdon et al., 2011). For a given sample, the *Ct* values were averaged between the two technical replicates and the relative FHV titre was calculated as Δ*Ct* = *Ct*_*RpL32*_ − *Ct*_*FHV*_.

### 2.7 Statistical analysis of survival data

Statistical analyses were performed in the r software (R Core Team 2013). Survival rates were analysed using Cox's proportional hazard mixed‐effect models (package coxme). This allowed estimating the antiviral protection conferred by each *Wolbachia* strain as a hazard ratio. The hazard ratio for a given *Wolbachia*‐infected line is the probability of death occurring at a given time point divided by the probability of death in the respective *Wolbachia*‐free line. Flies that were alive at the end of the experiment were treated as censored data.

To estimate the level of antiviral protection provided by each *Wolbachia* strain in the original hosts, we fitted the model:

(1) $$\lambda_{ijkl}=\lambda_{0}e^{H_{i}+W_{j}+H_{i}:W_{j}+v_{k}+\varepsilon_{ijkl}}$$

where λ_0_ is a baseline hazard, *H*_*i*_ is a fixed effect of host species *i*,* W*_*j*_ is a fixed effect of *Wolbachia* infection status *j* (infected or *Wolbachia*‐free), and *H*_*i*_:*W*_*j*_ is an interaction between host species and infection status. The vial in which each fly was found was treated as a random effect (*v*_*k*_) and ε_*ijkl*_ was the residuals.

The survival data in *D. simulans* STCP line were analysed with the simpler model:

(2) $$\lambda_{ikl}=\lambda_{0}e^{S_{i}+v_{k}+\varepsilon_{ikl}}$$

where *S*_*i*_ is the *Wolbachia* strain. The effect of *Wolbachia* in each host–*Wolbachia* association was tested using multiple pairwise comparisons (glht function, package multcomp, Hothorn, Bretz, & Westfall, 2008).

### 2.8 Statistical analysis of viral titres and *Wolbachia* density

*Wolbachia* density and viral titre data were analysed using a series of linear models. The *Wolbachia* density data were log‐transformed to reach the assumptions of normality and homoscedasticity. The effect of *Wolbachia* on viral titres in each host–*Wolbachia* association was further tested using multiple pairwise comparisons to compare the *Wolbachia*‐infected flies to the appropriate *Wolbachia*‐free control (glht function, package multcomp, Hothorn et al., 2008).

Viral titres (*T*) in the original host species were analysed as:

(3) $$T_{ijk}=H_{i}+W_{j}+H_{i}:W_{j}+\varepsilon_{ijk}$$

where the parameters are defined in Model (1). Viral titres (*T*) in *D. simulans* STCP were analysed as:

(4) $$T_{ik}=S_{i}+\varepsilon_{ik}$$

where the parameters are defined in Model (2). *Wolbachia* density (*D*) in *D. simulans* STCP and the original hosts was analysed as:

(5) $$D_{ik}=S_{i}+\varepsilon_{ik}$$

### 2.9 The relative importance of host and symbiont genomes

To quantify the relative importance of the host and symbiont genomes, we used an ANOVA to analyse trait data from both the original hosts and *D. simulans* STCP. The response variable was either relative survival (see below), relative viral titre (see below) or *Wolbachia* density of *Wolbachia*‐infected flies (*R*). This allowed us to fit the linear model:

(6) $$R_{ijk}=S_{i}/H_{j}+\varepsilon_{ijk}$$

In this data set, there is no cross‐factoring of the different hosts and symbiont strains. Therefore, we cannot distinguish a main effect of the host (an effect of the host on all *Wolbachia* strains) from a host‐by‐*Wolbachia* interaction (an effect of the host on specific *Wolbachia* strains). For this reason, the effect of the host *j* (*H*_*j*_) was nested within the effect of *Wolbachia* strain *i* (*S*_*i*_). As these were both treated as fixed effects, this is equivalent to fitting one main effect (*S*_*i*_) and one interaction (*S*_*i*_: *H*_*j*_).

For the survival data, the response variable *R*_*ijk*_ was the hazard of a vial of *Wolbachia*‐infected flies relative to the mean hazard of the *Wolbachia*‐free flies (*R*). This hazard ratio of each vial was estimated as a Best Linear Unbiased Predictor (BLUP) from a coxme model, with a separate model fitted to each host species. This model was identical to Model 2 except that the fixed effect was *Wolbachia* infection status (*W*) rather than strain (*S*).

For the viral titre data, the response variable *R*_*ijk*_ was the viral titre from each qPCR sample of *Wolbachia*‐infected flies relative to their respective *Wolbachia*‐free counterparts. This was calculated by normalizing each sample *i* to the average titre in the *Wolbachia*‐free controls as ΔΔ*Ct*_*i*_ = mean(Δ*Ct*_control_) − Δ*Ct*_Wolbachia *i*_. Here, mean(Δ*Ct*_control_) is the mean of all the *Wolbachia*‐free vials, and Δ*Ct*_Wolbachia *i*_ is the titre of *Wolbachia*‐infected sample *i*.

## 3 RESULTS

### 3.1 Symbiont‐mediated protection against viruses is common across natural *Drosophila*–*Wolbachia* associations

**Figure 1:**
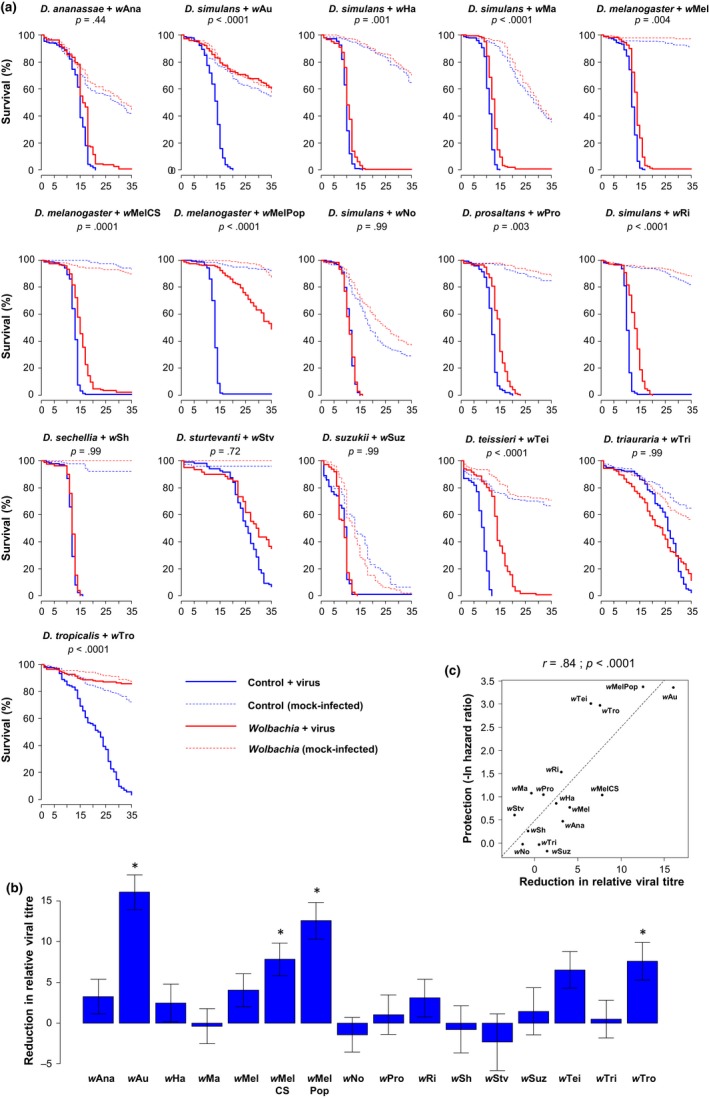
Antiviral protection in natural *Drosophila*–*Wolbachia* associations. (a) Survival curves following infection with FHV. *p*‐values indicate the significance of the difference between *Wolbachia*‐infected flies and their respective *Wolbachia*‐free counterparts (Model 1, see Methods). When this analysis was repeated on the mock‐infected flies, none of the *Wolbachia* strains significantly affected survival (Model 1; *p *> .05 in all cases). (b) *Wolbachia*‐induced reduction in viral titre calculated as the difference between *Wolbachia*‐free and *Wolbachia*‐infected flies. Positive values correspond to lower viral titres in *Wolbachia*‐infected flies on a log2 scale (ΔΔ*Ct*). Stars indicate significant differences between *Wolbachia*‐infected flies and their respective uninfected controls based on a multiple comparison test (Model 3, *p *< .05). Means, standard errors and *p*‐values were estimated from the Model 3 using the *glht* function to perform multiple comparisons. (c) Correlation between the increase in the survival of FHV‐infected flies caused by *Wolbachia* and the reduction in viral titre. The dashed line shows predicted values from a linear regression. *r* is Pearson's correlation coefficient between traits [Colour figure can be viewed at http://wileyonlinelibrary.com]

To assess how common *Wolbachia*‐mediated antiviral protection is, we tested whether a panel of 16 *Wolbachia* strains protected their natural host species against viral infection (Table 1). The 16 symbiont strains were in ten *Drosophila* species, and we created matched *Wolbachia*‐free lines. Following infection with the highly pathogenic RNA virus FHV, high rates of mortality were observed in all fly hosts (Figure 1a). The *Wolbachia* strains conferred varying levels of protection (Figure 1a; Model 1, host main effect: χ^2^ = 654.26, *df* = 30, *p* <* *.0001; *Wolbachia* main effect: χ^2^ = 396.06, *df* = 16, *p *< .0001; host‐by‐*Wolbachia* interaction: χ^2^ = 242.35, *df* = 15, *p* < .0001) with 10 of 16 *Wolbachia* strains significantly increasing the survival of their respective fly host after virus infection. While two *Wolbachia* strains prevented any virus‐induced mortality, many of the other protective strains only modestly increased survival (Figure 1a). The protective *Wolbachia* strains are found in five *Drosophila* species: *D. simulans*,* D. melanogaster*,* D. prosaltans*,* D. teissieri* and *D. tropicalis*. As found in previous studies (Chrostek et al., 2013; Martinez et al., 2014; Osborne et al., 2009), within *D. simulans* and *D. melanogaster*, different *Wolbachia* strains were associated with varying levels of protection against viruses.

The *Wolbachia* strains also varied in their effects on viral titre, measured as relative viral RNA copy number (Model 3, host main effect: *F*_15,95_ = 12.18, *p *< .0001; *Wolbachia* main effect: *F*_1,95_ = 43.07, *p *<* *.0001; host‐by‐*Wolbachia* interaction: *F*_15,95_ = 2.51, *p *=* *.004). Four of the symbiont strains significantly reduced titres (Figure 1b). Furthermore, the reduction in viral titre caused by *Wolbachia* was positively correlated with increases in survival after infection (Figure 1c). Overall, the change in titre explained 70% (*r* ^2^) of the variance in protection (Figure 1c).

### 3.2 Most variation in antiviral protection is explained by the symbiont strain rather than the host species

**Figure 2:**
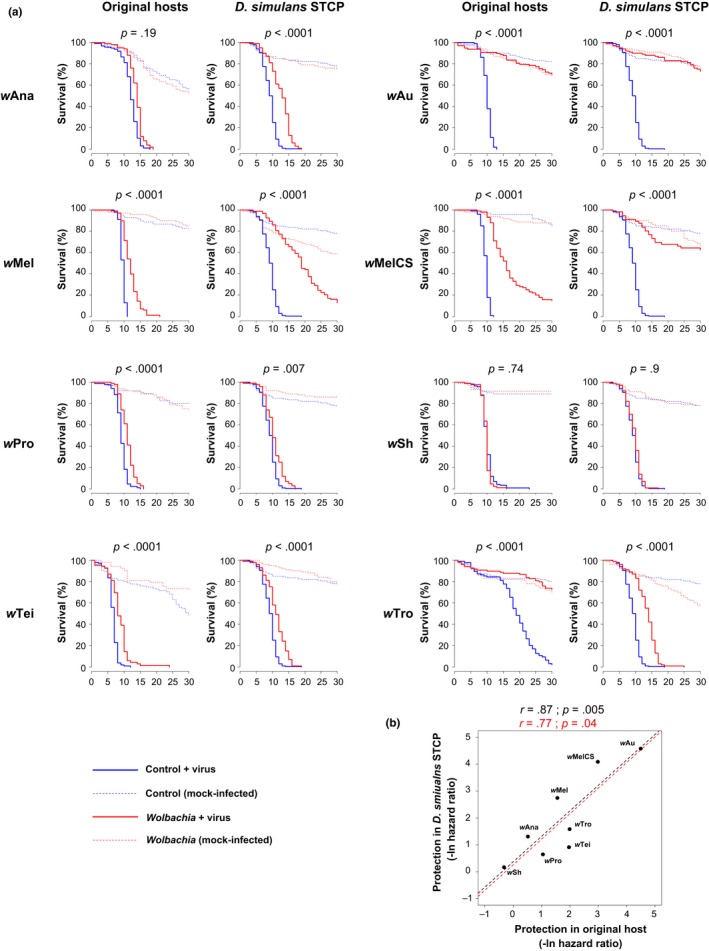
*Wolbachia*‐mediated protection in original hosts and a common genotype of *D. simulans*. (a) Survival curves following infection with FHV. *p*‐values for the comparisons of *Wolbachia*‐infected and *Wolbachia*‐free flies after infection with FHV are shown (Model 1 and 2, see Methods). When this analysis was repeated on the mock‐infected flies, none of the *Wolbachia* strains significantly affected survival (models 1 and 2; *p *> .05 in all cases). (b) Correlation in *Wolbachia*‐mediated increases in survival after FHV infection in the original hosts and the common genotype of *D. simulans*. The dashed lines show predicted values from linear regressions with (black) and without (red) *w*Au. *r* is Pearson's correlation coefficient between traits [Colour figure can be viewed at http://wileyonlinelibrary.com]

We next investigated the relative importance of the host genetic background and symbiont strain in determining whether *Wolbachia* protects its host against FHV. To this end, in a single experiment, we compared eight *Wolbachia* strains in both their original host species and a common genotype of *D. simulans* (STCP line, see Methods). As in the previous experiment, we followed fly survival upon infection with FHV and observed varying levels of protection among the original hosts as well as in the common genotype of *D. simulans* (Figure 2a). Both the *Wolbachia* strain and host background significantly affected the level of antiviral protection (Table 2a). However, while the *Wolbachia* strain explained more than 90% of the variance in protection, less than 5% was explained by symbionts behaving differently in the different hosts (Model 6, Table 2a). This suggests that antiviral protection mostly depends on the symbiont strain. In the case of the strain *w*Au, the original and new hosts are different genotypes of *D. simulans*. Because this could affect the correlation between the original and the new host, we ran the same analysis without *w*Au and found similar results (*Wolbachia* strain explains 91% of variation in protection). Accordingly, levels of protection were strongly correlated between the original hosts and the common genotype of *D. simulans* (Figure 2b), even when discarding *w*Au from the analysis (without *w*Au: *r *= .77; *p* = .04).

**Table 2:** Statistical analysis of *Wolbachia*‐mediated protection in original hosts and the common genotype of *D. simulans*

Trait^a^	Fixed effects^b^	*df*	Sum Sq	*F*‐values	*p*‐values	% variance explained
(A) Survival	*Wolbachia* strain	7	136.3	484.2	<.0001	93.6
	Host within *Wolbachia* strain	8	6.9	21.5	<.0001	4.7
	Residuals	61	2.5			1.7
(B) Viral titre	*Wolbachia* strain	7	920.1	49.6	<.0001	70.9
	Host within *Wolbachia* strain	8	172.9	8.2	<.0001	13.3
	Residuals	77	204.2			15.7
(C) *Wolbachia* density	*Wolbachia* strain	7	68.7	60.0	<.0001	81.2
	Host within *Wolbachia* strain	8	7.6	5.8	<.0001	8.9
	Residuals	51	8.3			9.9

a The survival response is the mean hazard of a vial of *Wolbachia*‐infected flies (20 flies in a vial) relative to the mean hazard of the *Wolbachia*‐free flies (see Methods). The viral titre and *Wolbachia* density responses were measured from pools of 10 flies.

b Described in Model 6 (see Methods).

**Figure 3:**
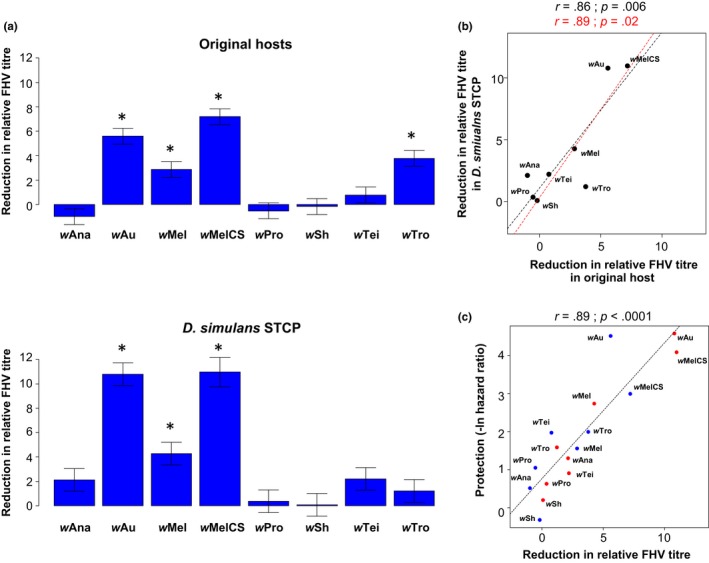
*Wolbachia*‐mediated reductions in viral titre in their original hosts and the common genotype of *D. simulans*. (a) Differences in the reduction in viral titre between *Wolbachia*‐free and *Wolbachia*‐infected flies. Positive values correspond to lower viral titres in *Wolbachia*‐infected flies on a log2 scale (ΔΔ*Ct*). Stars indicate significant differences between *Wolbachia*‐infected flies and their respective *Wolbachia*‐free controls based on a multiple comparison test (*p *< .05). Means, standard errors and *p*‐values were estimated using models 3 and 4 (see Methods). (b) Correlation between the effects of *Wolbachia* on FHV titre in original hosts and the common genotype of *D. simulans*. (c) Correlation between the effects of *Wolbachia* on FHV titre and survival after FHV infection. The blue and red points are the mean trait values per *Wolbachia* strain in the original host and the common genotype of *D. simulans*, respectively. In panels b and c, the dashed lines show predicted values from linear regressions with (black) and without (red) *w*Au and *r* is Pearson's correlation coefficient between traits [Colour figure can be viewed at http://wileyonlinelibrary.com]

We next examined the roles of host and symbiont genomes in determining the effect of *Wolbachia* on viral titres, and found similar patterns to our analysis of survival rates. *Wolbachia* had varying effects on viral titres, with 71% of the variation in our viral titre measurements being explained by the *Wolbachia* strain compared to the 13% explained by strains having different effects in different hosts (Model 6; Table 2b; Figure 3a, b). This was reflected in a strong correlation between the effect of *Wolbachia* on viral titre in the original hosts and the common genotype of *D. simulans* (Figure 3b), even if *w*Au is excluded (correlation without *w*Au: *r *=* *.89; *p* = .02).

The extent to which *Wolbachia* reduced viral titre was strongly correlated to increases in survival after FHV infection (Figure 3c). If the data from the common genotype of *D. simulans* and the original hosts are combined, changes in titre explain 80% of the variance in survival (Figure 3c; *r* ^2^ = .80). Furthermore, the strength of this correlation is similar if the data from the common genotype of *D. simulans* and the original hosts are analysed separately (original hosts: *r *=* *.83 and *p *=* *.01; common genotype of *D. simulans*:* r *=* *.96 and *p *=* *.0001).

### 3.3 Symbiont density is conserved when strains are transferred between host species and explains most of the variation in antiviral protection

**Figure 4:**
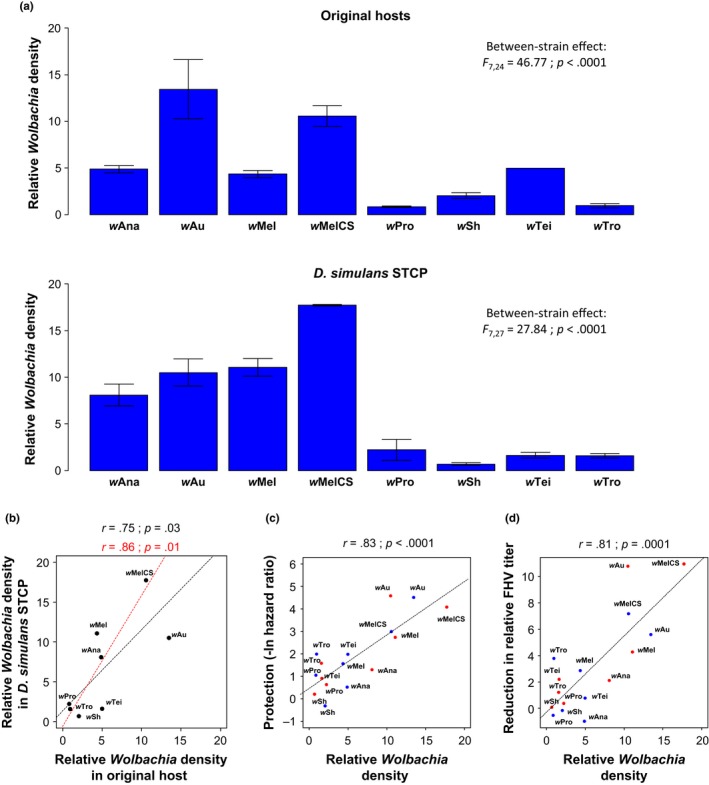
*Wolbachia* density and antiviral protection. (a) Mean *Wolbachia* density on a linear scale expressed as the copy number of the *Wolbachia* gene *atpD* relative to the fly gene *RpL32*. Between‐*Wolbachia* strain differences were tested using Model 5 on ln‐transformed data (see Methods). Error bars are standard errors. (b) Correlation in *Wolbachia* density between original hosts and the common genotype of *D. simulans*. (c) Correlation between the *Wolbachia*‐mediated increase in survival after FHV infection and *Wolbachia* density. (d) Correlation between the *Wolbachia*‐mediated reduction in FHV titre and *Wolbachia* density. The blue and red points are the mean trait values per *Wolbachia* strain in the original host and the common genotype of *D. simulans*, respectively. The dashed lines show predicted values from linear regressions with (black) and without (red) *w*Au and *r* is Pearson's correlation coefficient between traits [Colour figure can be viewed at http://wileyonlinelibrary.com]

Within a single host species, *Wolbachia*‐mediated protection is known to be tightly linked to the density of the symbiont in host tissues (Chrostek et al., 2013; Martinez et al., 2014; Osborne, Iturbe‐Ormaetxe, Brownlie, O'Neill, & Johnson, 2012; Osborne et al., 2009). To explain why the host genetic background has little effect on the level of antiviral protection that a given *Wolbachia* strain provides, we tested whether symbiont densities were conserved when a *Wolbachia* strain was moved between different hosts. We found significant variation in density between *Wolbachia* strains in both the original hosts and the common genotype of *D. simulans* (Figure 4a). As for protection, the *Wolbachia* strain explained far more of the variance in symbiont density than the host genetic background (Model 6; Table 2c), and there was a positive correlation between the density in the original hosts and the common genotype of *D. simulans* (Figure 4b; correlation excluding *w*Au: *r *= .86; *p *=* *.01). *Wolbachia* density was also correlated to the extent to which *Wolbachia* increased survival after viral infection (Figure 4c) as well as to the reduction in viral titre (Figure 4d). The strength of these correlations with symbiont density was similar in the original hosts and in the common genotype of *D. simulans* for both survival (original hosts: *r *= .81 and *p* = .01; *D. simulans*:* r* = .85 and *p *= .007) and reduction in viral titre (original hosts: *r *=* *.70 and *p *=* *.05; *D. simulans*:* r *=* *.86 and *p *=* *.006).

## 4 DISCUSSION

The extent to which *Wolbachia* protects insects against viruses varies greatly among host–symbiont associations. While symbiont strain is known to be a key determinant of protection (Chrostek et al., 2013; Martinez et al., 2014; Osborne et al., 2009), the role of the host genome has been poorly investigated. By comparing several *Wolbachia* strains in different host species, we found that the symbiont genome was far more important than the host genome in determining the level of protection. This was due to the density that a given *Wolbachia* strain reaches being conserved when it is moved to a new host.

In natural host–symbiont associations, we found that *Wolbachia* commonly protects *Drosophila* against viral infection. *Wolbachia* significantly decreased virus‐induced mortality in more than half (10/16) of the *Drosophila*–*Wolbachia* associations tested, although in most cases the increase in survival was only modest. This is similar to the patterns we have reported from a panel of *Wolbachia* strains that we transferred into *D. simulans*, where about half of the strains provided protection (Martinez et al., 2014). Other studies of single species found that *Wolbachia* protects against FHV in *Drosophila innubila* (Unckless & Jaenike, 2011), *D. suzukii* (Cattel et al., 2016) and *D. melanogaster* (Hedges et al., 2008; Teixeira et al., 2008), but not *D. bifasciata* (Longdon, Fabian, Hurst, & Jiggins, 2012). Unlike our results, interpreting these different studies can be difficult due to publication biases towards positive results and differences in experimental conditions and statistical power. For example, antiviral protection and symbiont density are affected by temperature and diet (Caragata et al., 2013; Mouton, Henri, Charif, Boulétreau, & Vavre, 2007; Serbus et al., 2015; Ulrich, Beier, Devine, & Hugo, 2016). This may explain why the strain *w*Ha was previously found to be nonprotective (Osborne et al., 2009) but conferred low levels of protection in our study using the same fly stock. Similarly, Cattel et al. (2016) found weak protection against FHV in *D. suzukii* but we did not.

By comparing the same symbionts in different hosts, we found that the symbiont strain was far more important than the host species in determining whether *Wolbachia* protects *Drosophila* against FHV. This was true in terms of both survival and viral titre. The large differences between *Wolbachia* strains have been reported before (Chrostek et al., 2013; Martinez et al., 2014; Osborne et al., 2009), but the small effect of the host was unexpected given that the host genetic background is critical to the expression of other *Wolbachia* phenotypes (Jaenike, 2007; Poinsot et al., 1998; Veneti et al., 2012).

As found in previous studies (Chrostek et al., 2013; Martinez et al., 2014; Osborne et al., 2009), the symbiont strains varied greatly in their densities and this correlated with antiviral protection. Critically, when the symbionts were transferred between host species, the strain‐specific densities were mostly conserved. Therefore, symbionts appear to regulate their density independently of the host, and this in turn determines the level of antiviral protection. Previous work found that the host genotype affects *Wolbachia* density (Kondo, Shimada, & Fukatsu, 2005; Mouton et al., 2007; Veneti et al., 2012). Our study does not contradict these results as we also found host effects on symbiont density, but these were small compared with the *Wolbachia* strain effect. Our results differ from studies of another *Drosophila* symbiont. As is the case for *Wolbachia*, the density of *Spiroplasma* symbionts that protect some *Drosophila* species against parasitic nematodes is similar between the native and the novel hosts (Haselkorn et al., 2013). However, the protective effect of different symbiont strains is decoupled from their density and strongly depends on the host species (Haselkorn et al., 2013). Therefore, the host genetic background is a strong determinant of the protective phenotype of *Spiroplasma* but not *Wolbachia*.

During its evolution, *Wolbachia* has frequently jumped between host species (Vavre et al., 1999; Werren et al., 1995; Zhang et al., 2013). Our results suggest the protective phenotype will often be transferred to the newly infected host. This could drive up the frequency of *Wolbachia* in the new host, potentially making protective strains more likely to move between species. This may be especially important for CI‐inducing *Wolbachia* strains, as these need to reach a minimum frequency in the population to be able to spread (Turelli, 1994). The benefit conferred by antiviral protection to the new host may promote the spread of the newly acquired *Wolbachia* infection allowing it to reach this threshold. The importance of this effect will depend on RNA viruses being a strong selective pressure, as highly protective *Wolbachia* strains are costly for the insect owing to their high density within host's tissues (Chrostek et al., 2013; Martinez et al., 2015). RNA viruses are extremely prevalent in *Drosophila* populations (Webster et al., 2015), but their effects on fitness in nature are unknown.

The observation that the host genome has comparatively little effect on antiviral protection or *Wolbachia* density is interesting. It seems likely that there is selection on hosts to control *Wolbachia* density to some optimal level, as RNA viruses are common *Drosophila* pathogens (Webster et al., 2015) and high *Wolbachia* densities substantially reduce host fitness (Martinez et al., 2015). Our finding that hosts have not evolved to modulate symbiont densities suggests there may be constraints that prevent flies from altering *Wolbachia* density. For example, *Wolbachia* may occupy an intracellular niche that protects it from insect immune defences (Bourtzis, Pettigrew, & O'Neill, 2000; Siozios, Sapountzis, Ioannidis, & Bourtzis, 2008). This could mean that hosts might be more likely to evolve tolerance to *Wolbachia* infections rather than mechanisms controlling the symbiont density.

Being able to predict the antiviral effects of a *Wolbachia* strain in a new host is useful for public health programmes that are releasing *Wolbachia‐*infected mosquitoes to prevent disease transmission. The Zika and dengue vector *Ae. aegypti* does not harbour *Wolbachia* in nature and therefore needs to be artificially infected with *Wolbachia* strains found in other host species (Hoffmann et al., 2015). These transfers are laborious and time‐consuming. Finding the optimal *Wolbachia* strains for disease control would be greatly facilitated by screening symbiont strains in *Drosophila* where the artificial transfer of *Wolbachia* between species has become routine (Chrostek et al., 2014; Martinez et al., 2014; Poinsot et al., 1998; Veneti et al., 2012). Our results suggest that such studies are likely to be a powerful way to select symbiont strains to be used as biocontrol agents.

We conclude that the extent to which *Wolbachia* protects different *Drosophila* species against viral infection depends primarily on the symbiont strain and not the host genome. This is due to *Wolbachia* regulating its density to similar levels in different host species. *Wolbachia* density in turn determines whether *Wolbachia* protects the host against viruses.

## AUTHOR CONTRIBUTIONS

J.M. and F.M.J. conceived the experiments. I.T., S.O., S.S., K.S., J.P.D. and J.M. conducted the experiments. J.M. and F.M.J. analysed the data and wrote the manuscript.

## DATA ACCESSIBILITY

The raw survival and qPCR data (FHV titre and Wolbachia density) are available at Dryad: https://doi.org/10.5061/dryad.869j5.
