# Supplementary material for: Symbiont strain is the main determinant of variation in Wolbachia‐mediated protection against viruses across Drosophila species
Source: Mol Ecol. 2017 May 30;26(15):4072–84. doi: 10.1111/mec.14164 (PMC5966720; doi:10.1111/mec.14164)
Supplement: Supplementary file 1 [file MEC-26-4072-s001.docx]

**Symbiont strain is the main determinant of variation in *Wolbachia*-mediated protection against viruses across *Drosophila* species**

Julien Martinez^1,2^, Ignacio Tolosana^1^, Suzan Ok^1^, Sophie Smith^1^, Kiana Snoeck^1^, Jonathan P Day^1^ and Frank Jiggins^1^

^1^Department of Genetics, University of Cambridge, UK

^2^Corresponding author ; email: jtm35@cam.ac.uk

**Table S1. List of *Drosophila* stocks used in this study**

| ***Drosophila* species** | ***Wolbachia* strain** | ***Drosophila* stock and references** |
| --- | --- | --- |
| *D. ananassae* | *w*Ana | 14024-0371.11 (San Diego Drosophila Species Stoch Center) (Mateos *et al.* 2006) |
| *D. melanogaster* | *w*MelCS | DrosDel *w*^1118^ iso (Chrostek *et al.* 2013) |
| *D. melanogaster* | *w*MelPop | DrosDel *w*^1118^ iso (Chrostek *et al.* 2013) |
| *D. melanogaster* | *w*Mel | DrosDel *w*^1118^ iso (Chrostek *et al.* 2013) |
| *D. sechellia* | *w*Sh | 14021-0248.08 (San Diego Drosophila Species Stoch Center) (Mateos *et al.* 2006) |
| *D. simulans* | uninfected | STCP, KB171 (Poinsot *et al.* 1998) |
| *D. simulans* | *w*Ha | DSH (O’Neill & Karr 1990) |
| *D. simulans* | *w*Ma | KB154A, provided by Kostas Bourtzis |
| *D. simulans* | *w*No | N7NO (Merçot & Poinsot 1998) |
| *D. simulans* | *w*Au | CO (Hoffmann *et al.* 1996) |
| *D. simulans* | *w*Ri | DSR (Hoffmann *et al.* 1986) |
| *D. simulans* | *w*Au | STCP[*w*Au] (Zabalou *et al.* 2008; Martinez *et al.* 2014) |
| *D. simulans* | *w*Ana | STCP[*w*Ana] (Zabalou *et al.* 2008; Martinez *et al.* 2014) |
| *D. simulans* | *w*MelCS | STCP[*w*MelCS] (Zabalou *et al.* 2008; Martinez *et al.* 2014) |
| *D. simulans* | *w*Mel | KB179 (Martinez *et al.* 2014) |
| *D. simulans* | *w*Sh | STCP[*w*Sh] (Zabalou *et al.* 2008; Martinez *et al.* 2014) |
| *D. simulans* | *w*Tei | STCP[*w*Tei] (Zabalou *et al.* 2008; Martinez *et al.* 2014) |
| *D. simulans* | *w*Pro | STCP[*w*Pro] (Zabalou *et al.* 2008; Martinez *et al.* 2014) |
| *D. simulans* | *w*Tro | STCP[wTro] (Zabalou *et al.* 2008; Martinez *et al.* 2014) |
| *D. teissieri* | *w*Tei | 14021‑0257.00 (San Diego Drosophila Species Stoch Center) (Mateos *et al.* 2006) |
| *D. triauraria* | *w*Tri | 14028-0651.00(San Diego Drosophila Species Stoch Center) (Mateos *et al.* 2006) |
| *D. suzukii* | *w*Suz | Provided by East Malling Research Station, collected in Italy |
| *D. prosaltans* | *w*Pro | WM19 (provided by Wolfgang Miller) |
| *D. sturtevanti* | *w*Stv | 14043-0871.10(San Diego Drosophila Species Stoch Center) (Mateos *et al.* 2006) |
| *D. tropicalis* | *w*Tro | 14030-0801.01(San Diego Drosophila Species Stoch Center) (Mateos *et al.* 2006) |

**Table S2. List of primers used in this study**

^a^published in (Longdon *et al.* 2011)

^b^designed on an exon-exon junction

**References**

Chrostek E, Marialva MSP, Esteves SS *et al.* (2013) Wolbachia Variants Induce Differential Protection to Viruses in Drosophila melanogaster: A Phenotypic and Phylogenomic Analysis. *PLoS Genetics*, **9**, e1003896.

Hoffmann A, Clancy D, Duncan J (1996) Naturally-occurring Wolbachia infection in Drosophila simulans that does not cause cytoplasmic incompatibility. *Heredity*, **76**, 1–8.

Hoffmann A, Turelli M, Simmons G (1986) Unidirectional incompatibility between populations of Drosophila simulans. *Evolution*, **40**, 692–701.

Longdon B, Hadfield JD, Webster CL, Obbard DJ, Jiggins FM (2011) Host phylogeny determines viral persistence and replication in novel hosts. *PLoS pathogens*, **7**, e1002260.

Martinez J, Longdon B, Bauer S *et al.* (2014) Symbionts Commonly Provide Broad Spectrum Resistance to Viruses in Insects: A Comparative Analysis of Wolbachia Strains. *PLoS Pathogens*, **10**, e1004369.

Mateos M, Castrezana SJ, Nankivell BJ *et al.* (2006) Heritable Endosymbionts of Drosophila. *Genetics*, **174**, 363–376.

Merçot H, Poinsot D (1998) Wolbachia transmission in a naturally bi-infected Drosophila simulans strain from New-Caledonia. *Entomologia Experimentalis et Applicata*, **86**, 97–103.

O’Neill SL, Karr TL (1990) Bidirectional incompatibility between conspecific populations of Drosophila simulans. *Nature*, **348**, 178–180.

Poinsot D, Bourtzis K, Markakis G, Savakis C (1998) Wolbachia Transfer from Drosophila melanogaster into D. simulans: Host Effect and Cytoplasmic Incompatibility Relationships. *Genetics*, **150**, 227–237.

Zabalou S, Apostolaki A, Pattas S *et al.* (2008) Multiple rescue factors within a Wolbachia strain. *Genetics*, **178**, 2145–60.
